# The Impact of Childhood Adversity on the Clinical Features of Schizophrenia

**DOI:** 10.1155/2015/532082

**Published:** 2015-08-04

**Authors:** Ravi Philip Rajkumar

**Affiliations:** Department of Psychiatry, Jawaharlal Institute of Postgraduate Medical Education and Research (JIPMER), Pondicherry 605 006, India

## Abstract

*Introduction*. Recent research has drawn attention to the link between childhood maltreatment and schizophrenia. Child abuse and neglect may have an impact on symptoms and physical health in these patients. This association has not been studied to date in India. *Materials and Methods*. Clinically stable patients with schizophrenia (*n* = 62) were assessed for childhood adversity using the Childhood Trauma Questionnaire. The association of specific forms of adversity with symptomatology and associated variables was examined. *Results*. Emotional abuse was reported by 56.5% patients and physical abuse by 33.9%; scores for childhood neglect were also high. Persecutory delusions were linked to physical abuse, while anxiety was linked to emotional neglect and depression to emotional abuse and childhood neglect. Physical abuse was linked to elevated systolic blood pressure, while emotional abuse and neglect in women were linked to being overweight. *Conclusions*. Childhood adversity is common in schizophrenia and appears to be associated with a specific symptom profile. Certain components of the metabolic syndrome also appear to be related to childhood adversity. These results are subject to certain limitations as they are derived from remitted patients, and no control group was used for measures of childhood adversity.

## 1. Introduction

In the past two decades, a growing body of research has called attention to the association between childhood adversity and psychotic disorders, particularly schizophrenia [1]. Patients with psychotic disorders have high rates of self-reported childhood abuse and neglect, ranging from 30% to over 75% [2–4]. This has led some authors to speculate that, in at least some cases of schizophrenia, childhood traumatic events play a causal role by affecting brain development, a “traumagenic neurodevelopmental model” [5]. Other researchers have focused on the role of specific types of childhood trauma, such as physical, emotional, or sexual abuse, in the development of specific symptoms such as auditory hallucinations [6–8] or delusions [8–10]. Along similar lines, preliminary evidence suggests that childhood abuse may be associated with positive psychotic symptoms, while neglect correlates more strongly with negative symptoms [11]. In addition to its effect on psychotic symptoms, childhood adversity may also be associated with components of the metabolic syndrome, such as dyslipidemia and elevated blood pressure, in patients with schizophrenia [12].

Despite the above evidence, the relationship between childhood adversity and schizophrenia is neither simple nor linear. Childhood adversity is neither necessary nor sufficient to cause schizophrenia [13], and a critical review of the association between childhood trauma and psychosis found only tentative evidence of a positive relationship, largely due to methodological problems with individual studies [14]. Moreover, childhood adversity is common both in other psychiatric disorders [15, 16] and in the general population [17], ruling out a one-to-one causal relationship. It is likely that childhood adversity may be associated with particular groups of symptoms, rather than with a categorical diagnosis such as schizophrenia [16], and that the association between childhood adversity and psychosis may be mediated by several factors. These include genetic variants [18, 19], the abuse of substances such as cannabis [20], ethnicity [21], gender [22], stressful life events in adulthood [23], and other environmental risk factors, such as urbanicity [24].

Most of the evidence linking childhood trauma and schizophrenia has been derived from studies in Western cultures. The impact of adversities during childhood in Eastern cultures, where parenting practices and social structures differ significantly from the West, may be different; however, there is preliminary evidence that rates of childhood trauma are elevated in Turkish [25] and Korean [26] patients with schizophrenia. To date, no study has examined the relationship between specific forms of childhood adversity and symptoms or symptom dimensions in schizophrenia in such a cultural setting.

In the current study, we examine the impact of different forms of childhood adversity—both abuse and neglect—on the clinical presentation of adult South Indian patients with a diagnosis of schizophrenia.

## 2. Materials and Methods

We screened all consecutive patients with a DSM-IV-TR diagnosis of schizophrenia who were being followed up at the out-patient unit of a general hospital psychiatry service in Pondicherry, South India. Of these patients, only those who had been stable on antipsychotic medication for the past six months, with no recent exacerbation of symptoms and with no psychiatric comorbidity, were included. Of the 139 patients screened, 62 fulfilled these criteria. Of the 77 patients excluded, seventy-four had experienced a psychotic episode in the six months prior to assessment. Further three were excluded due to a comorbid Axis I diagnosis, obsessive-compulsive disorder in two patients and social anxiety disorder in one. All patients provided written informed consent.

Information about the onset, duration, diagnostic subtype, symptom profile, and number of episodes of illness, as well as any medical or substance use comorbidity, was obtained from patients' medical records and was confirmed by interviewing patients and their accompanying caregivers. Systolic and diastolic blood pressure and height and weight were recorded during the interview, and the Body Mass Index (BMI) was calculated from this information.

Patients were assessed for the presence of various forms of childhood adversity using the Childhood Trauma Questionnaire (CTQ), a 28-item self-report instrument [27] which has been widely used to assess childhood trauma in patients with psychosis [4, 7, 8] and for which a Tamil adaptation is available [28]. Each item on the CTQ is scored from 1 to 5, and besides a total score, the instrument provides specific subscores for physical, emotional, and sexual abuse and for physical and emotional neglect.

Residual psychopathology was assessed using the Positive and Negative Syndrome Scale for Schizophrenia (PANSS) [28], and current depressive symptoms were assessed using the Calgary Depression Scale for Schizophrenia (CDSS) [29]. Patients' current level of functioning in various domains was assessed using the Functioning Assessment Short Test (FAST) [30] and the DSM-IV Global Assessment of Functioning (GAF) Scale [31].

Associations between the presence or absence of particular symptoms or illness variables and the CTQ total and subscores were assessed using the independent samples *t-*test or the Mann-Whitney *U* test, depending on the distribution of data. Correlations between the CTQ total and subscale scores and measures of psychopathology and functioning were examined using Pearson's *r* or Spearman's* rho*, again depending on the data distribution. All tests were two-tailed, and a value of *p* < 0.05 was considered significant.

## 3. Results

### 3.1. General Description of the Study Sample

The sample consisted of 62 patients, 31 men and 31 women. The mean age of the study subjects was 35.3 ± 8.5 years (range 19–54 years) and did not vary significantly by sex. 27 patients were single, 24 were currently married, 10 were divorced or separated from their spouses, and one was widowed; male patients were significantly more likely than women to be single (*χ* ^2^ = 10.57, *p* = 0.014). Patients had received an average of 9.9 ± 4.3 years of formal education (range 0–18 years), with no significant gender difference. Seventeen patients were unemployed, fourteen (all women) were homemakers, and the remainder were employed as skilled or unskilled labourers or farmers.

Description of the sample's clinical characteristics is provided in Table 1. Male and female patients were comparable in terms of PANSS symptom scores, CDSS-rated depression, and current levels of functioning and impairment. Male patients had higher rates of comorbid substance dependence, particularly nicotine (*χ* ^2^ = 4.77, *p* = 0.029), while female patients had a higher mean Body Mass Index (*t* = 2.184, *p* = 0.033).

### 3.2. Childhood Adversities Reported by Patients

The mean CTQ total and subscale scores are presented in Table 1. The highest scores were noted on the subscales for emotional neglect, followed by physical neglect and emotional abuse; scores on the physical and sexual abuse subscale scores were low. Overall, emotional abuse was reported by 35 (56.5%) patients, physical abuse by 21 (33.9%) patients, and sexual abuse by two (3.2%). Neither these categorical rates nor any of the CTQ subscores were significantly different across the sexes. We did not attempt a similar description of emotional or physical neglect, as almost all patients endorsed at least one item pertaining to these categories; besides, unlike sexual or physical abuse, neglect is difficult to categorize as a yes/no phenomenon. CTQ total and subscale scores did not differ according to place of childhood residence (rural or urban) or marital status and were not significantly correlated with total years of education.

### 3.3. Relationship between CTQ Scores and Clinical Course and Outcome

There was no difference in CTQ scores between patients with an acute onset of schizophrenia and those with an insidious onset; however, the CTQ Physical Abuse subscore was significantly higher in those patients (*n* = 53) whose illness was not triggered by a life event or stressor (Mann-Whitney *U* = 144.0, *p* = 0.025). Age at onset, total duration of illness, illness course, and duration of untreated psychosis were not correlated with any of the CTQ scores. CTQ scores did not differ according to the course of the illness (episodic versus continuous), and CTQ scores in remitted patients did not differ from those who had significant residual symptoms; these findings held true for both men and women. There was no significant relationship between the GAF or FAST scores and any of the CTQ scores, and this remained true even after controlling for sex.

### 3.4. Relationship between CTQ Scores and Symptomatology

The PANSS positive, negative, general psychopathology, and total scores did not correlate significantly with the CTQ total or subscale scores; this association remained negative in both men and women. When the presence or absence of individual symptoms was used as a grouping variables, the CTQ Physical Abuse subscore was significantly associated with lifetime delusions (*t* = 2.89, *p* < 0.01), and more specifically with delusions having a persecutory theme (*t* = 2.81, *p* < 0.01). The presence of auditory hallucinations, disorganization, catatonic symptoms, and first-rank schizophrenic symptoms was not associated with a significant difference in any CTQ scores. Similarly, the presence or absence of individual negative symptoms—affective flattening, asociality, alogia, attentional impairment, and anhedonia—was not associated with CTQ abuse, neglect, or total scores. However, when we examined only the 31 patients with significant negative symptoms and a diagnosis of residual schizophrenia, the CTQ Physical Neglect subscore was positively correlated with the PANSS negative symptom score (Pearson's *r* = 0.364, *p* = 0.048). When categorical definitions of physical, emotional, and sexual abuse were used in the analysis, no specific psychotic symptom or PANSS score was specifically associated with any individual form of abuse.

### 3.5. Relationship between CTQ Scores and Other Illness Variables

The total CTQ score was associated with lifetime suicide attempts in male patients, but not in women (*t* = 2.11, *p* = 0.043); similarly, the CTQ Physical Neglect score was associated with violent suicide attempts, but only in men (*t* = 2.47, *p* = 0.02). Lifetime substance use disorders were not associated with elevated CTQ scores irrespective of gender.

The PANSS anxiety scale score was significantly correlated with the CTQ Emotional Neglect subscore (Spearman's *ρ* = 0.253, *p* = 0.049), and this remained significant even after correcting for gender. The PANSS depression score was positively correlated with the CTQ Emotional Neglect (*ρ* = 0.278, *p* = 0.03), Physical Neglect (*ρ* = 0.271, *p* = 0.035), and total (*ρ* = 0.27, *p* = 0.036) scores. Similarly, the CDSS total score was positively correlated with the CTQ Emotional Abuse, Emotional Neglect, Physical Neglect, and total scores (*p* < 0.01 for all correlations except Physical Neglect, *p* = 0.022), and mean CDSS scores were significantly higher in those with lifetime emotional abuse (*t* = −2.44, *p* = 0.018).

Systolic and diastolic blood pressures were not correlated with CTQ scores in either men or women. However, the CTQ Emotional Abuse subscore was positively correlated with patients' Body Mass Index (BMI) (Pearson's *r* = 0.335, *p* < 0.01). When using a cut-off value of 25 kg/m^2^ for the BMI, patients whose BMI was above 25 had significantly higher total CTQ scores (*t* = 2.84, *p* < 0.01) and higher subscores for emotional abuse (*t* = 3.15, *p* < 0.01), emotional neglect (*t* = 2.53, *p* = 0.014), and physical neglect (*t* = 2.23, *p* = 0.03); an analysis of covariance revealed that this association was not mediated by exposure to atypical antipsychotics (*F* = 0.05, *p* = NS); however, there was a significant effect of gender, with differences remaining significant in female, but not in male patients. Mean systolic blood pressure was higher in those reporting lifetime physical abuse (mean 120.86 mmHg versus 114.53 mmHg in those without physical abuse; *t* = 2.12, *p* = 0.038).

## 4. Discussion

The rates of physical and emotional abuse reported by our patients are comparable to those reported in the literature [2, 3]; however, rates of reported sexual abuse are much lower. While this may represent a genuine difference, it is far more likely to reflect underreporting on the part of patients. This is probably due to the cultural taboos and stigma that surround child sexual abuse in the Indian context [32], as a survey conducted by the Government of India suggested that up to 42% of a sample of 2211 children had experienced some form of sexual abuse [33]; moreover, higher rates of childhood sexual abuse in patients with psychosis are well-documented in other settings [34, 35]. Measures of the different types of abuse and neglect did not differ according to gender; this is in accordance with a recent meta-analysis which found that the various forms of childhood maltreatment, except for sexual abuse, were equally common in male and female patients with schizophrenia [2].

The onset of schizophrenia was slightly more likely to be spontaneous, that is, to occur in the absence of an obvious proximal stressor, in those with higher scores on the Physical Abuse subscale. This is consistent with the hypothesis that childhood physical abuse represents a form of “social defeat” which can itself predispose to psychosis, by causing cognitive changes and/or altered dopamine transmission [36]. The latter alteration may involve a gene/environment (G × E) interaction in which genetic variants which affect frontal dopamine levels interact with physical abuse to increase the likelihood of developing psychotic symptoms [18]. Though we did not find a relationship between outcome measures and childhood adversity in our sample, this result is unlikely to be significant as our patients were already selected for relative clinical and functional stability.

Among the different types of psychotic symptoms reported by patients, only delusions—particularly those with a persecutory theme—were significantly associated with physical abuse. This result is consistent with work indicating that child victims of physical abuse are likely to experience impaired reality testing [37] and that physical abuse can lead to the development of paranoid ideation [38] or a paranoid personality [39]. Similarly, a study of patients with schizophrenia has also reported an association between physical abuse and positive psychotic symptoms such as delusions [10]. Variables that may mediate this association include variants of the catechol-O-methyltransferase gene [18], anxiety [38], and negative beliefs about the self or others [38, 40]. We could not replicate the often-reported association between childhood sexual abuse and auditory hallucinations [6–8, 21], most probably because of the underreporting of sexual abuse in our sample. We found some evidence of an association between negative symptoms and physical neglect, as suggested by earlier literature [11], but this result must be interpreted with caution as it was derived from a subgroup analysis.

Besides delusions, childhood adversity was associated with a range of other nonpsychotic symptomatology, depression, anxiety, and suicidal behavior. The latter association was more marked in men, while the others remained true irrespective of gender. Further, similar associations were obtained regardless of which depression measure—the ordinal PANSS depression score or continuous CDSS total score—was used. In other words, there was good concurrent validity for the association between depression and childhood adversity—particularly childhood neglect—in patients with schizophrenia. This is consistent with the results of the Genetic Risk and Outcome of Psychosis (GROUP) investigators, who suggest that childhood trauma is linked to a cluster of symptoms—depression, anxiety, and positive psychotic symptoms—which transcends existing diagnostic categories or groups [16, 41]. An association between childhood abuse and suicide risk in schizophrenia has been reported in earlier studies [3, 10], though our finding that male gender may mediate this association requires further exploration.

Finally, we found evidence of an association between specific forms of abuse and components of the metabolic syndrome. First, physical abuse was linked to elevated systolic blood pressure. The latter relationship has already been observed in patients with schizophrenia [12] and is unlikely to be an artefact of the interview conditions, as blood pressure was measured before any enquiries were made about childhood trauma. Second, female patients who were overweight reported significantly more emotional abuse and neglect during childhood. A link between childhood maltreatment and obesity has been demonstrated in the general population [42] and in patients with mood disorders [43] and has been shown to be mediated by gender [44]. However, this association has not been examined so far in schizophrenia. In our patients, this finding could not be explained by antipsychotic exposure and is likely to involve both biological and psychological mechanisms [45, 46].

Our results are subject to certain limitations. First, the sample size was small compared to similar work in other populations. Second, we relied on a single self-report measure of childhood adversity, which may have resulted in an underreporting of the scope of this problem. Third, owing to the small number of patients in each group, we may have underestimated the associations between specific psychotic symptoms and childhood adversity. Fourth, as some of the information was collected retrospectively, it is subject to recall bias. Fifth, our sample included only clinically stable patients. While this might have led to a loss of information, it was necessitated by the strictures of the institute's Ethics Committee against administering the potentially stressful CTQ to acutely ill, unstable patients. Sixth, we excluded a small number of patients with comorbid diagnoses, in order to clearly delineate the relationship between childhood adversity and schizophrenia alone; however, these patients did not differ significantly from the rest of the sample in terms of CTQ scores. Finally, due to the preliminary and exploratory nature of our work, our study lacked a control group, and we are therefore unable to comment on whether the patients in our sample experienced higher levels of abuse and neglect than appropriately matched controls from the same population. It is hoped that the latter deficit, which is admittedly the greatest shortcoming of our study, will be addressed by a continuation of our work which is currently in progress and involves the recruitment of a control sample.

## 5. Conclusion

In spite of these shortcomings, our results replicate many of the positive findings in the schizophrenia literature on the impact of childhood adversity. They are consistent with the view that childhood maltreatment, despite not being necessary to cause schizophrenia, can have a significant impact on symptomatology. To our knowledge, this report is the first study from India to explore the association between childhood adversity and schizophrenia. From a clinical perspective, our findings highlight the need to actively enquire about childhood maltreatment in patients with schizophrenia, particularly those who are overweight or who present with symptoms of anxiety or depression even after improvement in positive psychotic symptoms.

## Figures and Tables

**Table 1:** Clinical description of the sample by gender.

Variable	Male patients (*n* = 31)	Female patients (*n* = 31)	Total
Age at onset, years	24.9 ± 7.4	26.6 ± 7.9	25.8 ± 7.6
Duration of illness, years	10.8 ± 8.6	8.3 ± 7.2	9.6 ± 7.9
Duration of untreated illness, years	1.4 ± 1.8	1.5 ± 1.5	1.5 ± 1.7
Diagnostic subtype			
Paranoid	22	22	44
Undifferentiated	7	5	12
Catatonic	2	2	4
Disorganized	—	2	2
Body mass index, kg/m^2^	23.2 ± 4.2	25.9 ± 5.4	24.5 ± 4.9^*∗*^
Blood pressure, mmHg			
Systolic	118.3 ± 10.3	115.1 ± 12.4	116.7 ± 11.4
Diastolic	80.9 ± 10.2	77.5 ± 10.2	79.2 ± 10.2
Lifetime substance use disorder			
Total	10	3	13^*∗*^
Nicotine dependence	10	3	13^*∗*^
Alcohol abuse/dependence	2	0	2
Lifetime suicide attempt	9	8	17
Lifetime violent suicide attempt	5	4	9
CTQ scores			
Emotional abuse	7.9 ± 3.9	8.0 ± 3.3	8.0 ± 3.6
Physical abuse	6.4 ± 2.9	5.7 ± 1.9	6.1 ± 2.4
Sexual abuse	5.0 ± 0.2	5.1 ± 0.4	5.0 ± 0.3
Emotional neglect	11.6 ± 3.5	11.5 ± 3.3	11.5 ± 3.4
Physical neglect	9.3 ± 2.7	9.6 ± 3.3	9.5 ± 3.0
Total	40.4 ± 10.4	39.9 ± 10.1	40.1 ± 10.2
PANSS scores			
Positive	8.9 ± 2.2	8.9 ± 2.1	8.9 ± 2.2
Negative	15.4 ± 6.4	15.5 ± 6.3	15.5 ± 6.3
General psychopathology	24.5 ± 6.6	26.6 ± 7.5	25.6 ± 7.1
Total	48.8 ± 13.7	51.0 ± 14.4	49.9 ± 14.0
CDSS score, total	1.3 ± 2.3	1.5 ± 2.7	1.4 ± 2.5
FAST score			
Autonomy	2.9 ± 2.6	3.8 ± 2.8	3.4 ± 2.7
Occupational activity	7.2 ± 4.8	7.7 ± 4.6	7.4 ± 4.7
Cognitive functioning	4.6 ± 3.0	5.2 ± 3.0	4.9 ± 3.0
Finances	1.8 ± 1.6	1.9 ± 1.8	1.9 ± 1.7
Interpersonal relationships	7.0 ± 4.9	8.6 ± 5.5	7.8 ± 5.2
Leisure	1.9 ± 1.6	2.5 ± 1.7	2.2 ± 1.6
Total	25.4 ± 16.7	29.6 ± 17.4	27.5 ± 17.0
GAF score	61.7 ± 11.5	58.9 ± 11.6	60.2 ± 11.5

PANSS: Positive and Negative Symptom Scale for Schizophrenia; CDSS: Calgary Depression Scale for Schizophrenia; CTQ: Childhood Trauma Questionnaire; FAST: Functional Assessment Short Test; GAF: Global Assessment of Functioning.

^*∗*^Difference is significant at *p* < 0.05.
